# HEALTHCARE-ASSOCIATED INFECTION AND UNFAVOURABLE OUTCOMES DURING A ONE-YEAR FOLLOW-UP AFTER DISCHARGE: A SINGLE-CENTER STUDY

**DOI:** 10.13075/ijomeh.1896.02473

**Published:** 2025

**Authors:** Beata Czerniak, Wioletta Banaś, Jacek Budzyński

**Affiliations:** Nicolaus Copernicus University in Toruń, Ludwik Rydygier Collegium Medicum in Bydgoszcz, Department of Vascular and Internal Medicine, Toruń, Poland

**Keywords:** risk factors, healthcare-associated infection, functional status, readmission, long-term prognosis, single-centre study

## Abstract

**Objectives:**

Healthcare-associated infections (HAIs) are persistent problem in contemporary in-hospital patients' treatment but they are recognized as potentially preventable. The influence of HAI on patients' outcomes after discharge is not fully acknowledged. The authors conducted the study to determine the associations between HAI and length of hospitalization, all-cause in-hospital death, need for treatment in the intensive care unit (ICU), and rehospitalization within 14 days, 30 days, and 365 days.

**Material and Methods:**

On the basis of inclusion criteria, 631 of 5322 (11.86%) inpatients were enrolled to the study, for whom the authors determined, among other factors, medical history, *Activities of Daily Living* (ADL) score and *Nutritional Risk Screening 2002* (NRS-2002) score, nutritional status (using anthropometric characteristics and bioelectrical impedance analysis), and *Charlson Comorbidity Index* score.

**Results:**

Healthcare-associated infections occurred in 17.9% of the inpatients enrolled to the study. Healthcare-associated infections were linked with greater length of in-hospital stay (LOS), risk of in-hospital death, transfer to the ICU, and hospital readmission within 14 days and 30 days. In multivariate analysis, HAI was the strongest risk factor for LOS prolongation, need for treatment in the ICU (OR = 15.26, 95% CI: 3.0–77.8, p < 0.01), and all-cause in-hospital death (OR = 10.73, 95% CI: 3.9–29.69, p < 0.001), alongside NRS-2002 and ADL scores. Healthcare-associated infections did not affect the risk of 14- and 30-day and 1-year readmissions in multivariate analysis, which were related to, among other factors, ADL score and mode of admission.

**Conclusions:**

Healthcare-associated infections statistically and significantly affected only outcomes related to the current hospitalization, across both univariate and multivariate analyses.

## INTRODUCTION

Healthcare-associated infections (HAIs) are defined as the occurrence of infection symptoms 48 h after admission or during the 90 days following the patient's discharge. They are a serious and persistent problem in contemporary health care, affecting, on average, 5.6–10% of inpatients in Poland, which means that approx. 400 000 patients acquire HAI in 1 year [1]. Despite improvements in the effectiveness of HAI therapy, HAIs still lead to the prolongation of patients' in-hospital stay, a rise in hospitalization costs, increased morbidity and in-hospital mortality (e.g., by 0.7% for urinary tract infection and by 10.2% for hospital-acquired pneumonia) [2–7]. However, there are few data concerning the influence of HAI on the risk of early (14and 30-day) and long-term (1-year) unplanned hospital readmissions. Moreover, hospital readmissions are considered an indicator of healthcare quality, increased risk related to hospitalization per se (e.g., thromboembolic complications, HAI, immobilization, isolation, and falls), and are associated with greater healthcare resource utilization, which, in accordance with the regulations in Poland, is not reimbursed by the health insurer.

Worldwide, the rate of all-cause 30-day readmissions ranges to 10–25% [8–16]. Among several factors listed as potential causes of readmission, both preventable (e.g., inadequate diagnostics and treatment) and non-preventable (e.g., frailty, unavoidable chronic disease progression, comorbidities, and patients' non-adherence to treatment [10,17–20]), HAIs are listed as potentially preventable factors [10]. Therefore, the authors conducted the study to determine the associations between the occurrence of HAI and “non-infectious” endpoints, i.e., length of hospital stay, occurrence of in-hospital death, need for the patient's transfer to the intensive care unit (ICU), and rehospitalization within 14 days, 30 days, and 365 days. Moreover, the authors also compared the statistical power of the influence of HAI on patients' 1-year mortality and the need for rehospitalization with other clinical factors (e.g., demographic factors, nutritional status, and comorbidities).

## MATERIAL AND METHODS

### Patients

Of the 5322 patients hospitalized in the clinic of the authors' university hospital in January 2017 – December 2018, 631 (11.86%) were enrolled to the study. The inclusion criteria were as follows: hospitalization in the clinic, a medical record that had been assigned an even number, and informed consent to participate in the study. The study group consisted of 262 (41.5%) women and 369 (58.5%) men, all of whom were white, and aged 18–98 years (mean [M] ± standard deviation [SD] 67.10±14.20 years). So small percentage of patients enrolled resulted from either patient's refusal to take part in the study or their inability to sign conscious consent to participate in the study.

### Methods

The study was performed in accordance with an observational, cohort, prospective study design. All patients included in the study completed a survey questionnaire compiled by the authors, provided a medical history, and underwent a physical examination. Anthropometric parameters of nutritional status assessment were collected for all patients, and included: weight, height, BMI, waist, arm, and calf circumference, handgrip strength, and clinically driven biochemical determinations. Body composition was assessed by bioelectrical impedance analysis (BIA) (using a TANITA BC-420 device, TANITA Corporation, Tokyo, Japan, in line with European Union Medical Device Directive MDD 93/42/EEC). The following assessments were also undertaken for each patient:

– functional status, using scores on the *Activities of Daily Living* (ADL) scale and *Norton Scale* (a scoring system for assessing risk of bedsores);
– malnutrition risk, using the score on the *Nutritional Risk Screening 2002* (NRS-2002) scale [21];
– HAI risk, using the authors' hospital's current survey form;
– score on a comorbidity scale, employing the age-adjusted *Charlson–Deyo Comorbidity Index* (CCI) [22,23];
– and scores on the *Cumulative Illness Rating Scale* (CIRS) [24] and ATLAS (i.e., age, temperature, leukocytes, albumin, systemic antibiotics given for >1 day) scale [25].

The use of so many heath status measures were used to check their predictive power for HAI risk and the risk of “non-infectious” outcomes measured, including readmissions rate. The analysis of clinical usefulness of respective indices for HAI risk was performed in the other publication [26].

Healthcare-associated infections occurrence was defined as the occurrence of infection symptoms 48 h after admission [1]. Only HAI occurred during current hospitalization were taken into account.

### Outcomes measured

Shortand long-term outcomes were determined for all study patients. Short-term outcomes were those that occurred during the current hospitalization: length of in-hospital stay (LOS), the need for transfer to the ICU, and in-hospital death. The long-term outcomes examined were all-cause readmission within 14 days and 30 days and 1 year after discharge, which were determined during remote follow-up based on medical records or telephone interviews at several time intervals, that is, after 14 days, 30 days, 90 days, and 365 days.

### Ethics

The study was carried out in compliance with consent No. KB 705/2016 given by the local Bioethics Committee and the written consent of each study participant.

### Statistics

Statistical analysis was conducted using the licensed version of the statistical analysis software STATISTICA v. 13.1 (TIBCO Software, Inc., Palo Alto, USA, 2017). The results were presented as the M±SD or as a frequency (n, %) of the categorical variables. The statistical significance level was set at a p-value of <0.05. The normal distribution of the study variables was analysed using the Kolmogorov-Smirnov test. The statistical significance of differences between groups was verified using the Student's t-test and, for the categorical variables, the χ2 test. Multiple regression for determining factors influencing LOS, and logistic regression was used for the calculation of odds ratios (ORs) and 95% confidence intervals (CIs), both in unifactorial and multifactorial analyses.

## RESULTS

The demographic and clinical characteristics of the patients studied are presented in relation to HAI occurrence, which amounted to 17.9% of the inpatients enrolled to the study (Table 1). As expected, the occurrence of HAI prolonged the length of hospitalization (by approx. 5 times) and increased the prevalence of in-hospital death (Table 2); moreover, it increased the risk of in-hospital death, of transfer to the ICU, and of hospital readmission within 14 days and 30 days (Table 3). The rehospitalization rate among all 631 patients amounted to 2.2%, 4.3%, and 13% within 14 days, 30 days and 1 year after discharge, respectively (Table 2). Of those rehospitalizations, 50.0%, 37.04%, and 17.07% were related to HAI, respectively. However, it should be underlined that only 6.2%, 8.9%, and 12.4% of patients discharged after HAI treatment required rehospitalization within 14 days, 30 days and 1 year, respectively.

**Table 1.** Clinical characteristics of patients with and without healthcare-associated infection (HAI), hospitalized in January 2017 – December 2018, Department of Vascular and Internal Medicine, Toruń, Poland

Variable	Participants (N = 631)		p
	with HAI (N = 113, 17.9%)	without HAI (N = 518, 82.1%)	
Demographic			
age [years] (M±SD)	73.68±15.78	65.68±13.44	<0.001
male gender [n (%)]	65 (57.52)	304 (58.69)	0.820
Medical			
urgent admission [n %)]	103 (91.15)	297 (57.34)	<0.001
history of hospitalizations during 12 months prior hospitalization [n (%)]	66 (58.41)	263 (50.77)	0.141
BMI [kg/m^2^] (M±SD)	27.59±6.44	27.50±5.47	0.915
waist-to-hip ratio (M±SD)	0.93±0.09	0.96±0.09	0.546
fat mass [% (% of whole body mass)]	18.00 (15.97)	28.88 (10.32)	0.038
fat-free mass [kg] (M±SD)	54.65±7.42	54.43±11.58	0.969
visceral fat level [pts] (M±SD)	10.50±9.47	12.31±5.54	0.520
handgrip strength [kg] (M±SD)	35.90±13.27	28.85±11.43	0.173
bedsore at admission [n (%)]	20 (17.70)	4 (0.77)	<0.001
*Norton Scale* [pts] (M±SD)	12.74±4.86	18.74±2.75	<0.001
*Nutritional Risk Screening 2002* [pts] (M±SD)	2.01±1.38	0.85±1.04	<0.001
*Activities of Daily Living* at admission [pts] (M±SD)	2.21±2.45	5.48±1.41	<0.001
*Cumulative Illness Rating Scale* [pts] (M±SD)	16.60±5.44	11.39±5.48	<0.001
ATLAS [pts] (M±SD)	5.22±1.44	2.65±1.67	<0.001
*Charlson Comorbidity Index* [pts] (M±SD)	5.70±3.20	3.87±2.66	<0.001
steroid therapy [n (%)]	27 (23.89)	24 (4.63)	<0.001
blood transfusion [n (%)]	54 (47.79)	59 (11.39)	<0.001
leg amputation [n (%)]	12 (10.62)	4 (0.77)	<0.001
creatinine [mg/dl]	1.62±1.12	1.53±6.44	0.886
white blood cell count [G/l] (M±SD)	17.55±48.84	9.22±9.82	<0.001
hemoglobin [g/l] (M±SD)	11.44±2.34	13.20±4.69	<0.001
red blood cells [T/l] (M±SD)	3.89±0.77	4.36±0.84	<0.001
hematocrit [%] (M±SD)	34.90±6.52	38.84±6.93	<0.001
red cell distribution width [%] (M±SD)	16.38±2.49	14.93±2.42	<0.001
procalcitonin [ng/ml] (M±SD)	3.96±14.10	5.46±15.96	0.556
C-reactive protein [mg/dl] (M±SD)	103.06±123.77	53.25±90.17	<0.001

ATLAS – age, temperature, leukocytes, albumin, systemic antibiotics given for >1 day.

**Table 2.** Prevalence of in-hospital outcomes measured in relation to healthcare-associated infection (HAI) occurrence in the patients hospitalized in January 2017 – December 2018, Department of Vascular and Internal Medicine, Toruń, Poland

Variable	Participants (N = 631)		p
	with HAI (N = 113, 17.9%)	without HAI (N = 518, 82.1%)	
In-hospital stay [days] (M±SD)	20.07±26.57	4.24±4.06	<0.001
Intensive care unit treatment [n (%)]	16 (14.16)	3 (0.58)	<0.001
All-cause in-hospital death [n (%)]	43 (38.05)	8 (1.54)	<0.001
Readmission after discharge [n (%)]			
within 14 days	7 (6.19)	7 (1.35)	0.002
within 30 days	10 (8.85)	17 (3.28)	0.008
within one year	14 (12.39)	68 (13.13)	0.833

**Table 3.** Healthcare-associated infection (HAI) as a risk factor for the occurrence of the outcomes measured in the patients hospitalized in January 2017 – December 2018, Department of Vascular and Internal Medicine, Toruń, Poland

Variable	Healthcare-associated infection (OR (95% CI))	p
All-cause in-hospital death	30.49 (13.02–71.42)	<0.001
Necessity for intensive care unit treatment	28.32 (8.07–99.28)	<0.001
Readmission after discharge		
within 14 days	4.82 (1.65–14.06)	<0.001
within 30 days	2.86 (1.27–6.44)	0.01
within one year	0.94 (0.51–1.73)	0.083

In the next part of the analysis, the authors tried to compare the power of the influence of HAI and other clinical factors on the occurrence of the outcomes measured using multiple regression for LOS (Table 4) and multivariate logistic regression for need for treatment in the ICU, all-cause in-hospital death, and readmission occurrence (Table 5). A statistically significant multiple regression model with an R^2^ coefficient that amounted to 0.30 showed that the LOS was related independently to patients' age, nutritional risk (NRS-2022 score), functional status (ADL score), CIRS score, CCI score, blood C-reactive protein concentration, white blood cell count, and HAI occurrence (Table 4). Of those factors, HAI occurrence had the highest β and partial correlation coefficient values. Surprising, neither parameters of nutritional status assessment (both anthropometric and those obtained by BIA) nor biochemical determinations affected either the short-or long-term clinical outcomes during the 1-year observation period.

**Table 4.** Multiple regression model for length of in-hospital stay in the patients hospitalized in January 2017 – December 2018, Department of Vascular and Internal Medicine, Toruń, Poland

Variable	β	Standard β error	t(612)	p
Constant			1.01	0.314
Demographic				
age	–0.154	0.041	–3.71	<0.001
male gender	–0.047	0.042	–1.11	0.266
Medical				
admission mode (urgent/planned)	0.024	0.037	0.66	0.511
healthcare-associated infection occurrence	0.309	0.047	6.53	<0.001
BMI	–0.038	0.052	–0.74	0.460
fat mass percentage of whole body mass	0.006	0.052	0.12	0.903
fat-free mass	0.053	0.059	0.90	0.371
waist circumference	0.022	0.058	0.38	0.705
handgrip strength	–0.006	0.042	–0.14	0.887
*Norton Scale*	0.085	0.073	1.16	0.247
*Nutritional Risk Screening 2002*	0.160	0.044	3.66	<0.001
*Activities of Daily Living*	–0.226	0.074	–3.05	0.002
ATLAS	0.009	0.040	0.22	0.825
*Cumulative Illness Rating Scale*	0.130	0.058	2.25	0.025
*Charlson Comorbidity Index*	–0.116	0.054	–2.17	0.031
C-reactive protein	0.166	0.037	4.49	<0.001
hemoglobin	0.027	0.036	0.77	0.444
albumin	–0.004	0.034	–0.12	0.906
white blood cell count	–0.082	0.039	–2.12	0.034
creatinine	0.039	0.035	1.12	0.262

ATLAS – age, temperature, leukocytes, albumin, systemic antibiotics given for >1 day.

R = 0.55, R^2^ = 0.30, F(18, 612) = 14, 725, p < 0.001.

**Table 5.** Risk factors for occurrence of outcomes measured in multifactorial analysis using logistic regression (per unit of variable) in the patients hospitalized in January 2017 – December 2018, Department of Vascular and Internal Medicine, Toruń, Poland

Variable	ICU treatment	All-cause in-hospital mortality	Readmission		
			14-day	30-day	1-year
Age					
OR	1.00	0.99	1.04	1.00	1.02
95% CI	0.97–1.04	0.98–1.03	0.98–1.10	0.97–1.03	0.99–1.04
p	0.80	0.97	0.97	0.62	0.13
Admission mode					
OR	2.11	0.77	1.25	1.31	0.36
95% CI	0.24–18.77	0.23–2.56	0.29–5.33	0.47–3.64	0.21–0.62
p	0.66	0.66	0.76	0.61	<0.001
*Nutritional Risk Screening 2002*					
OR	1.66	1.45	0.82	0.92	1.09
95% CI	1.17–2.35	1.08–1.94	0.45–1.48	0.61–1.37	0.84–1.39
p	<0.001	0.013	0.51	0.68	0.54
*Norton Scale*					
OR	0.99	1.01	0.75	0.81	0.91
95% CI	0.83–1.19	0.89–1.14	0.61–0.91	0.69–0.95	0.80–1.03
p	0.96	0.85	<0.01	<0.01	0.14
*Activities of Daily Living* (ADL)					
OR	0.84	0.64	1.80	1.45	1.30
95% CI	0.58–1.22	0.49–0.83	1.07–2.99	1.01–2.10	0.97–1.72
p	0.36	<0.001	0.026	0.049	0.074
ATLAS					
OR	0.64	1.15	1.26	1.10	0.88
95% CI	0.42–0.99	0.86–1.53	0.78–2.02	0.76–1.60	0.69–1.13
p	0.046	0.35	0.35	0.59	0.32
*Cumulative Illness Rating Scale* (CIRS)					
OR	1.13	0.96	1.02	1.00	0.96
95% CI	0.95–1.33	0.86–1.06	0.88–1.19	0.91–1.11	0.90–1.04
p	0.16	0.77	0.79	0.94	0.37
*Charlson Comorbidity Index* (CCI)					
OR	0.68	0.99	1.02	1.12	0.98
95% CI	0.48–0.96	0.85–1.18	0.79–1.35	0.94–1.34	0.84–1.13
p	0.03	0.96	0.96	0.89	0.76
Healthcare-associated infection					
OR	15.26	10.73	2.39	1.62	2.04
95% CI	3.0–77.83	3.9–29.69	0.49–11.7	0.49–5.31	0.84–4.96
p	<0.01	<0.001	0.28	0.42	0.11

ATLAS – age, temperature, leukocytes, albumin, systemic antibiotics given for longer than 1 day; ICU – intensive care unit.

The authors obtained also statistically significant logistic regression equations for all the outcomes measured (Table 5). Healthcare-associated infections was the strongest risk factor for the need for treatment in the ICU (together with NRS-2002, ATLAS, and CCI scores), and all-cause in-hospital death (together with NRS-2002 and ADL scores). However, HAI did not affect the risk of 14-day, 30-day and 1-year readmissions, which were related mainly to patients' functional status at admission (expressed by *Norton Scale* and ADL scores), and mode of admission (1-year readmission risk).

## DISCUSSION

In the authors' observational, prospective, cohort study, the authors evaluated the prevalence and predisposing factors for in-hospital endpoints and readmission within 14 days and 30 days and 1 year after discharge. The authors confirmed that HAI occurrence affected not only the short-term outcomes of inpatients, using both univariate and multivariate analyses (Table 3–5), but univariate analysis also revealed that HAI also increased the risk of readmission within 14 days and 30 days of discharge (Table 3). In the multivariate analysis, the authors found that HAI maintained its independent effect only in regard to the outcomes related to the current hospitalization (e.g., LOS) (Table 4) and was not a significant factor in relation to the risk of hospital readmission, which was related to functional status (expressed by *Norton Scale* and ADL scores) and mode of admission (Table 5). It was surprising that need for readmission was not influenced by parameters of nutritional risk or status assessment, or by comorbidity burden (Table 5).

The authors' observations concerning the adverse influence of HAI occurrence and NRS-2002, ADL, and CCI survey scores on the risk of in-hospital mortality and need for ICU treatment (Table 2) corroborate well-known data published by other authors [2–7]. What was surprising was the negative correlations between LOS and CCI score and white blood cell count (Table 4), because it is known that comorbidity burden is a potentially important factor in increasing the risk of LOS prolongation and unfavourable outcomes among inpatients [9,15,23].

The authors' results concerning hospital readmissions only partially corroborate reports published by other authors. In the authors' study, the readmission rate (Table 2) was relatively small (4.3%) in relation to the findings from other research in which, for example, the 30-day readmission rate amounted to 2.61–25% [8–16,20,27], and in meta-analysis of 24 publications of the 1.5 million individuals with heart failure the 30-day readmission rate amounted to 13.2% (95% CI: 10.5–16.1%) [16]. However, in the authors' large sample analysis of consecutive hospitalizations in years 2014–2015, 30-day readmission rate in the same hospital amounted to 6.23% [21], and in the other Polish hospitals, Kryś et al. [9] found a 30-day all-cause readmission rate of 12.5%, two-thirds of these readmissions (8.33%) were classified as unplanned. In another study, concerning Polish patients with newly diagnosed heart failure in years 2013–2019, 30-day unplanned heart failure readmission rate was 2.96% [15]. In the authors' study, the authors analysed the risk of only non-scheduled rehospitalizations. However, due to several factors that are particular to each country (e.g., hospitalization cost reimbursement and population and hospital density), direct comparisons of hospital readmission rates among publications from different countries (such as those that differ in terms of income and healthcare levels) and study periods (e.g., 2010–2018, due to progress in treatment effectiveness and safety) would seem to be strongly biased and methodologically unjustified.

In Bianco et al. [11] and Balane et al. [10], about 43% of hospital readmissions were recognized as potentially avoidable, which makes them a potential target for health-care-quality improvement efforts [11]. In the studies by Belane et al. [10] and Dreyer and Viljoen [8], >40% of potentially preventable hospital 30-day readmissions were related to HAI, which is similar to the authors' analysis, in which 50%, 37.04%, and 17.07% of 14-day, 30-day and 1-year readmissions were related to HAI, respectively. However, in the authors' study, only 6.19%, 8.85%, and 12.39% of patients discharged after HAI treatment required rehospitalization within 14 days, 30 days or 1 year, respectively. This may explain why in univariate analysis HAI occurrence increased the risk of 14and 30-day hospital readmission by 4.82 and 2.86 times, respectively (Table 3), and in multivariate analysis using logistic regression HAI occurrence was superseded by *Norton Scale* and ADL scores in predicting 14and 30-day hospital readmissions (Table 5). Paradoxically, in contrast to the observations of other authors [8–15,27–29], in the authors' study, CCI score did not have a significant influence on the risk of hospital readmission (Table 5). Maybe, the score of Readmission Risk Assessment Tool (RRAT), consisting of 8 modifiable risk factor categories: *Staphylococcus aureus* colonization, tobacco use, obesity (BMI), cardiovascular disease, venous thromboembolic disease, neurocognitive/psychological/behavioral problems, physical deconditioning, and diabetes [30], might be better predictor of all cause readmission in internal medicine ward, however the authors did not use this tool.

The results the authors obtained should be interpreted in the context of a few limitations. First, the patients were not enrolled to the study through a standard method of randomization; the patient selection was done on the basis of patients having been assigned a medical record with an even number of medical histories. Moreover, a significant percentage of patients who had originally qualified did not consent to take part in the study. Second, the authors did not analyse respective types of HAI (e.g., urinary tract infection or bloodstream infection) due to their relatively low number, which made it impossible to evaluate associations between HAI types and the outcomes measured. The relatively low number of HAIs also meant that the authors did not analyse the relationships between the outcomes measured and respective causes of hospitalization and diagnosis at discharge. Third, the authors also did not analyse the number of medications per day recommended for patients at discharge, despite it being known that the use of more than 5–10 medications is associated with increased probability of preventable readmissions [11,17]. Fourth, an evaluation of the association between HAI and readmission risk can potentially be biased due to the subjective nature of decisions by physicians working in emergency departments (EDs). Moreover, physicians' decisions may be affected by the large number of people attending EDs [9], which is typical of the way these departments function in Poland.

## CONCLUSIONS

Healthcare-associated infections was statistically significantly found to prolong hospitalization and increase the risk of in-hospital all-cause mortality, transfer to the ICU, and rehospitalization within 14 days and 30 days of discharge. However, the influence of HAI on the occurrence of the outcomes measured in multivariate analysis remained independent only in regard to the current hospitalization-related outcomes; the risk of readmission was related independently only to patients' functional status at admission and mode of admission. This shows, that during every hospitalisation all in-patients should be stratified according to the risk of HAI, and preventive action should be applied to decrease the length of hospitalization and all-cause in-hospital death. Moreover, among in-patients with low scores in ADL scale and *Norton Scale* the physiotherapy and mobilization programs introduced during hospitalization and extended for out-patient time with supervision using, e.g., tele-medicine ought to be more widely used in order to prevent readmissions.
